# CytoSaLPs score: a promising new tool for the detection and screening of extrauterine high grade serous carcinoma

**DOI:** 10.1186/s12885-023-10607-w

**Published:** 2023-02-16

**Authors:** Sofia Lekka, Victoria Psomiadou, Theodoros Panoskaltsis, Eleni Tsouma, Natasa Novkovic, Helen Trihia, Olympia Tzaida, Dimitrios Korfias, Panagiotis Giannakas, Christos Iavazzo, Panagiotis Vakas, Nikolaos Vlahos, George Vorgias

**Affiliations:** 1 Department of Gynecology, Metaxa Memorial Cancer Hospital, Piraeus, Greece; 2 2nd Department of Obstetrics and Gynecology, National Kapodistrian University of Athens, Aretaieion Hospital, Athens, Greece; 3 Department of Pathology, Metaxa Memorial Cancer Hospital, Piraeus, Greece

**Keywords:** ovarian cancer, tubal cytology

## Abstract

**Background:**

A recent theory supports that high-grade serous epithelial ovarian cancer derives from the fimbrial end of the fallopian tube and during the last decade, a few studies have examined the fallopian tube cytology. Our study aims to determine the cytomorphological characteristics of both benign and non-benign salpingeal samples, in order to establish fallopian cytology as a valuable diagnostic test for women with high risk for development of ovarian/fallopian/peritoneal cancer.

**Methods:**

Our study included patients undergoing salpingoophorectomy or total hysterectomy and salpingoophorectomy for any gynecological pathology. Using a soft brush, fallopian tube smears from the fimbrial end were collected ex vivo. The Cytologists of our Institution described the morphological characteristics of the fallopian cells by adopting a proposed Table, which had a calibration system ranging from 3 to 29. This Table is referred to as the *CytoSaLPs Score.* Our study compared the two diagnostic cytological methods, the one of the conventional cytology and the other using the *CytoSaLPs Score*, having as gold standard the tubal’s pathological findings.

**Results:**

A total of 230 tubal specimens from 144 patients were included in this study. The *Score’s* mean for the benign and non-benign arm was 12.8 and 18.7 respectively. The cut-off point for both arms was 16.5. The *CytoSaLPs Score* tool showed significantly higher specificity (87.50% vs. 75.96, p-value < 0.001) and positive predictive value PPV (40.91% vs. 26.47%, p-value < 0.001) compared to conventional cytology. Regarding the accuracy, the Score’s superiority is highlighted (86.96% vs. 76.52%, p-value < 0.001).

**Conclutions:**

The evaluation of tubal cytology using the *CytoSaLPs Score* could be used as a reliable diagnostic method. Further evaluation with larger studies is warranted.

**Supplementary Information:**

The online version contains supplementary material available at 10.1186/s12885-023-10607-w.

## Introduction

Ovarian cancer is the leading cause of death among gynecological malignancies. In the United States alone, it is estimated that in 2020, 21,750 newly diagnosed cases and 13,940 deaths occurred Siegel, Miller [1]. Unfortunately, 60% of the patients are diagnosed at an advanced stage, reaching a 5-year survival rate of 29%, while on the contrary, the 5-year survival rate for early-stage patients is approximately 92% [2]. This high mortality rate coming as a result from the combination of delayed diagnosis due to the late onset of symptoms and the lack of an effective screening test.

Throughout the literature, epithelial ovarian cancer is often used as an umbrella term for ovarian, fallopian tube and primary peritoneal carcinomas [3]. It is known that 90% of ovarian malignancies consist of epithelial types of ovarian cancer, among which the high-grade serous carcinoma (HGSC) is considered to be the most aggressive. Recent pathologic, genomic and molecular evidence implicate the fimbriae end of the fallopian tube as the origin of the majority of high-grade tumors [4]. Further evidence also indicates that other histologic types, including low-grade carcinomas, may also derive from the fallopian tube [5, 6]. Thus, more precise examination and direct sampling of the fallopian tube could be the key for earlier detection of these types of cancer.

In this study, we introduce the *CytoSaLPs Score*, which is a calibration point system ranging from 3 to 29 based on the morphological characteristics of the fallopian tubes’ cells. Our study evaluated smears collected ex vivo from the fimbriae end of the fallopian tube, using a soft brush, from patients undergoing salpingoophorectomy or total hysterectomy and salpingoophorectomy for any gynecological indication. Our purpose is to assess the diagnostic value, as well as to determine the effectiveness of the *Score* as an early diagnostic and predictive method for extrauterine high-grade serous carcinomas.

## Methods

The study was approved by the Institutional Review Board of “Metaxa” Memorial Cancer Hospital and written informed consent was obtained from each patient. The enlisted patients were recruited from the Gynecologic Oncology Department of our Institution from April 2020 to May 2021. A total of 144 patients undergoing salpingoophorectomy or total hysterectomy and salpingoophorectomy for any gynecological indication were included in our study. Demographic and clinical information was collected including age, indication for surgery, type of surgical procedure, body mass index (BMI), levels of CA-125 and HE-4.

**Fig. 1:**
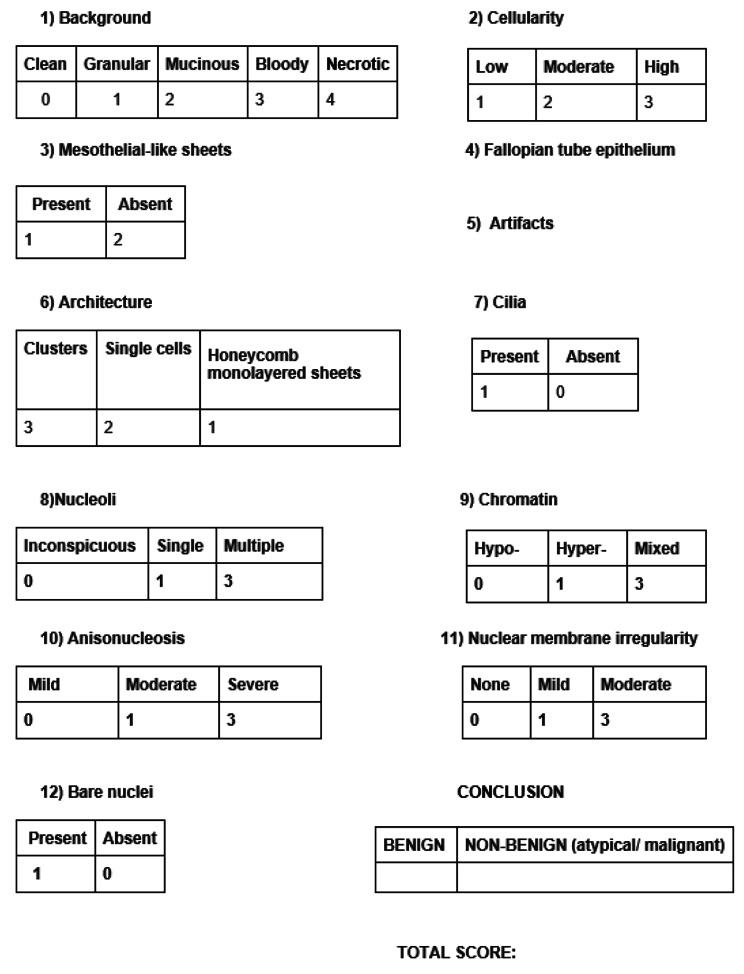
The CytoSaLPs Score

Cytological samples were collected by introducing a soft cytobrush into the fimbriae and rotating it forward clockwise into the non-fimbrial part of the salpinx until it reached the isthmus. Finally, the brush was pulled backwards through a slow (< 1 cm/s), counterclockwise rotation. Subsequently, the sample was coated and fixated on a slide, followed by rinsing the brush in a container with preservative solution, which was sealed and marked with the patient’s details. The container remained stored at 4 °C, until the sample was placed in CytoLyt® liquid material and examined cytologically with the Pap stain. The samples were collected ex vivo, immediately after the removal of the specimen. The cytological analysis was performed by the Cytologists of our Institution, who were unaware of the surgical findings or the final pathology report. All samples were identified by a unique ID number. Using both solid-based and liquid-based cytology, the Cytologists described the morphological characteristics of the fallopian tube cells. Furthermore, we compared our cytological findings to those of previous studies that described fallopian tube cell lesions [7, 8] and we formed a calibration system from 3 to 29 evaluating specific fallopian tube cell characteristics and summarizing them into a novel calibrating system, which is referred to as the CytoSaLPs Score tool (Fig. 1).

**Fig. 2:**
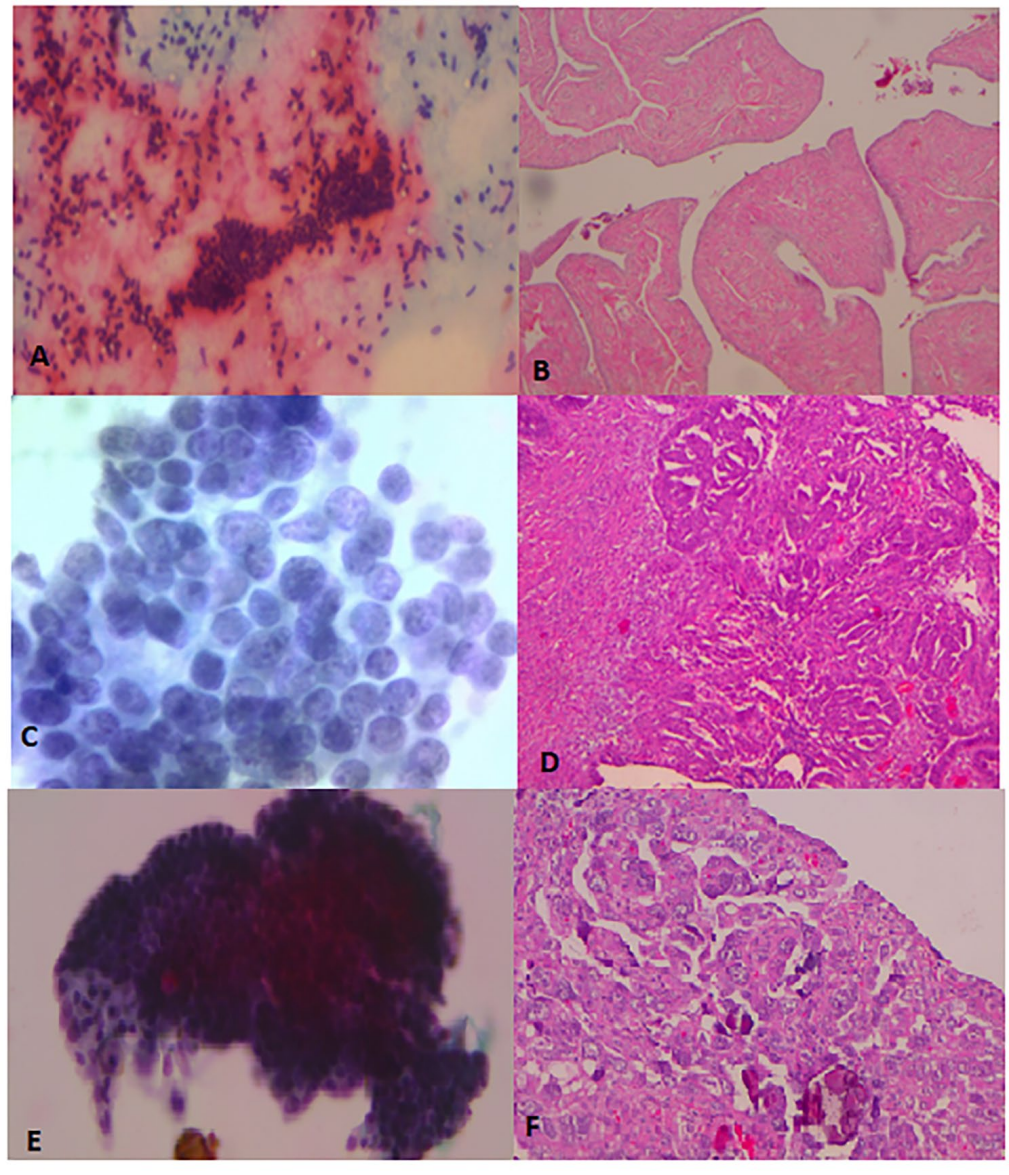
Cytological evaluation of fallopian tube cells: comparison with pathologic findings **A**) Pap-stain x10: monolayer sheet of cells with hyperchromatic chromatin and bare nuclei in the background (benign) **B**) A&H x25: Hyperplasia of the tubal epithelium (benign) **C**) Thin prep x40: Granular abnormal distribution of chromatin, presence of small nucleolus, normal contour of the nuclear membrane (atypia). **D**) A&H x100: Cribriform architectural pattern and moderate cellular atypia (STIC) **E**) Thin prep x20: complex cluster with the presence of psammoma bodies (malignant) **F**) A&H x250: Complex (focally solid) architectural pattern and severe cellular atypia and abundant psammoma bodies (malignant)

The cytologic report was based on two different methodologies. The first one categorized the samples as benign and non-benign (atypical/malignant) based on conventional cytology. The second one was based on the use of the *CytoSaLPs Score* tool and characterized the samples according to an interval scale (Fig. 2). Our study compared the two diagnostic cytological methods having as gold standard the SEE-Fim protocol applied in all fallopian tube pathology specimens [9].

### Statistical analysis

The Shapiro Wilk test was used to estimate the normality of data distribution. Correlation between two continuous variables was assessed using Pearson’s correlation coefficient or Spearman’s rank correlation coefficient accordingly. The two tailed Student’s t-test or Mann-Whitney U-test were performed, as appropriate, to compare mean values between the two groups. The optimal cut-off point of the *CytoSaLPs Score*, in order to subgroup results in benign and non-benign arms, was identified using both the ROC curve and the Youden index. Specificities and sensitivities were based on the optimal cut-off point.

Data’s statistical analysis was performed using both SPSS (25th edition) and MedCalc Software Ltd [10]. To consider a result as statistically significant, p-value ought to be less than 0.05.

## Results

A total of 230 smears were evaluated from 144 patients with an average age of 56.5 (± 13.6) and an average BMI of 28.4 (± 5.5). The mean Ca-125 level was 245 (± 1649.3) and the mean HE-4 level 166.9 (± 326.3). Cytological samples were obtained from both the right and left fallopian tubes in 117 patients. 27 patients had single tubal cytological testing either because they had a contralateral salpingectomy or adnexectomy in their medical history or in cases where the fimbriae occluded, or unilateral salpingectomy was performed due to desire for childbearing or wish of the patient. The salpingectomy-indication was set in the setting of ovarian cancer (7.6%), adnexal mass (46.6%), endometrial hyperplasia or cancer/ EIN (20.1%), endometrial mass- leiomyomas or persistent polyps (20.1%), uterine prolapse (1.4%) and cervical cancer or dysplasia (4.2%). We performed 6 salpingoophorectomies (4.1%), 88 total hysterectomy with bilateral salpingoophorectomy (61.1%), 13 total hysterectomy with bilateral salpingoophorectomy plus omentectomy/omental biopsy (9%), 34 explorative laparotomies including total hysterectomy with bilateral salpingoophorectomy, pelvic lymph node dissection and para-aortic lymph node dissection, peritonectomy, enterectomy, splenectomy, focal hepatectomy, appendectomy (23.7%) and 3 radical total hysterectomy with bilateral salpingoophorectomy (2.1%). Final pathology report documented 60 malignant cases out of 144 patients. More specifically, 30 cases of extrauterine pelvic carcinomas were identified (22 originating from the ovaries, 4 from the fallopian tube and 2 from the peritoneum), 27 cases of endometrial cancer and 3 cases of cervical cancer.

Of the specimens obtained from the 144 patients included in our study, thirty-one were evaluated as non-diagnostic, either because the sample lacked cellularity, or because it was inadequate and therefore were excluded from our study. Moreover, cytologic evaluation of the smears in our study yielded to identify the malignant lesion/infiltration in 18 out of the 22 fallopian tubes that were diagnosed with cancer or malignant infiltration overall, including one specimen of serous tubal intraepithelial neoplasia (STIC). These cases concerned HGSC ovarian cancer, HGSC fallopian cancer, 1 case of cervical cancer and 3 cases of serous endometrial cancer.

**Fig. 3:**
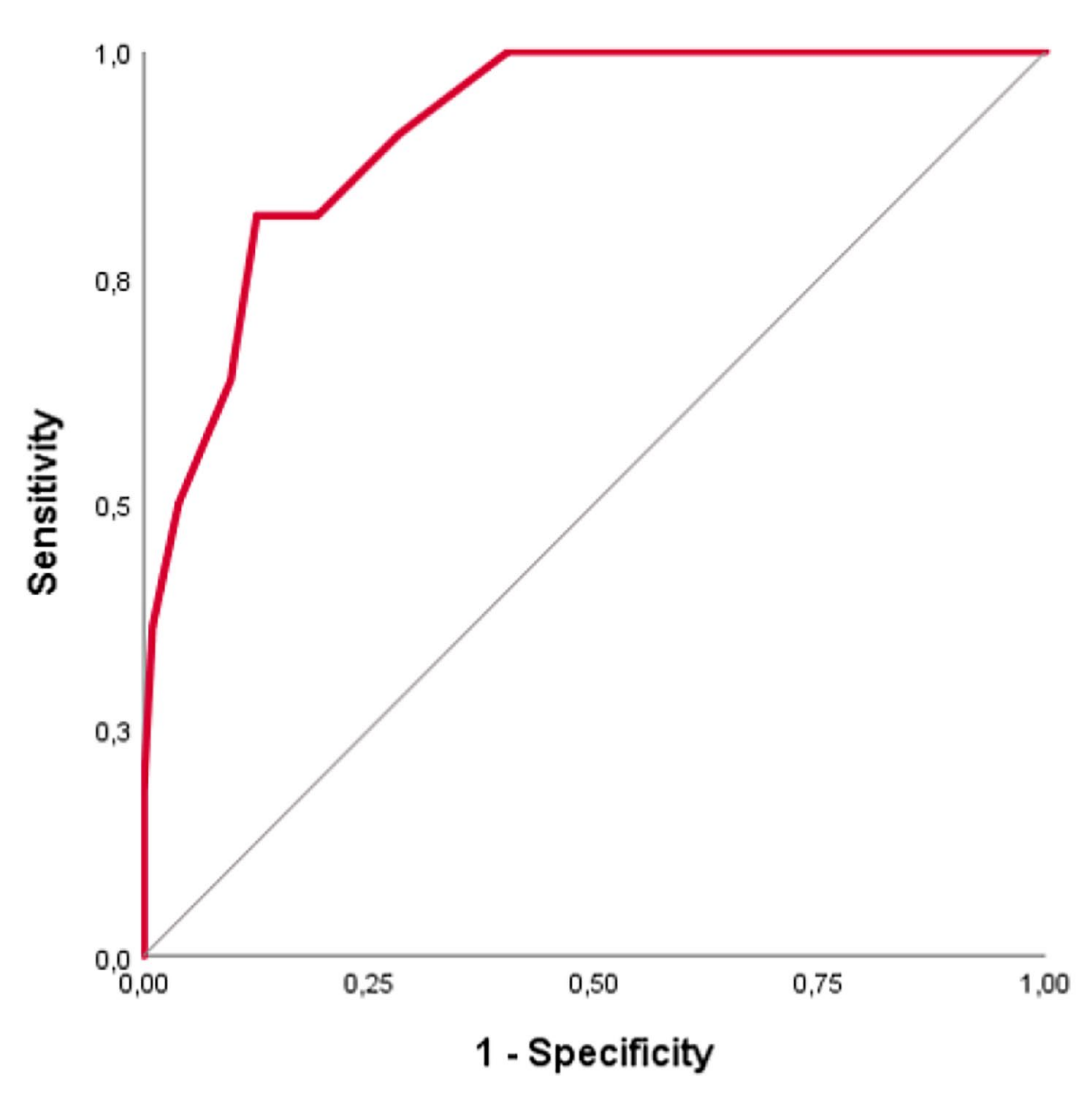
The Roc Curve

Based on histopathological findings, the patients were separated in two subgroups, the benign and the non-benign arm. A crosstabulation was conducted so that 230 samples could be matched to a histopathological diagnosis and therefore eligible for further analysis. The *Score’s* mean ± SD for the benign and non-benign arm is 12.8 (± 3.1) and 18.7 (± 3.0) respectively. Moreover, the *Score’s* median for non-benign arm is higher than the one of the benign arm (18.5 vs. 12.5). According to the Youden index, the cut-off point for benign and non-benign arm is 16.5. Thus, in detail, it is indicated that the salpingeal smear of a patient with a *CytoSaLPs Score* lower than 16.5, is probably benign. Otherwise, smears scoring higher than 16,5 are evaluated as non-benign (atypical or malignant). Using the cut-off point (16.5) and the ROC curve (Fig. 3), the Area Under the Curve (AUC) for *CytoSaLP Score* is 0.914 (95% CI: 0.864–0.964, p-value < 0.001), indicating this suggested *Score* as a reliable diagnostic tool.

Both approaches presented the same sensitivity of 81.82% (95% CI = 59.72 – 94.81%). However, comparing the diagnostic ability of the cytological approaches, we found notable, statistically significant differences. Specifically, the *CytoSaLPs Score* tool showed significantly higher specificity (87.50% vs. 75.96, p-value < 0.001) and positive predictive value PPV (40.91% vs. 26.47%, p-value < 0.001) compared to conventional cytology. In contrast, Score’s negative predictive value (NPV) showed a modest improvement, without statistical significance (97.53% vs. 97.85%). However, regarding the accuracy of the two cytological methods, the Score’s superiority is highlighted (86.96% vs. 76.52%, p-value < 0.001). Based on the aforementioned results, it is illuminated that the *CytoSaLPs Score* tool is of great diagnostic ability. Table 1 summarizes the diagnostic values for both approaches.

**Table 1:** Diagnostic values for both cytological approaches [10]

Value	Conventional cytology approach	CytoSaLP Score approach	p-value
Sensitivity	81.82% (95% CI = 59.72 – 94.81%)		
Specificity	75.96% (95% CI = 69.57 – 81.60%)	87.50% (95% CI = 82.22 – 91.67%)	< 0.001
Positive predictive value	26.47% (95% CI = 20.86 – 32.96%)	40.91% (95% CI = 31.48 – 51.06%)	< 0.001
Negative predictive value	97.53% (95% CI = 94.19 – 98.97%)	97.85% (95% CI = 94.93 – 99.10%)	n.s.
Accuracy	76.52% (95% CI = 70.50 − 81.84%)	86.96% (95% CI = 81.91 − 91.02%)	< 0.001

## Discussion

There is a growing interest in the detailed assessment of the fallopian tubes, arising from a recent theory regarding the carcinogenesis of malignant serous ovarian tumors. Identification of fallopian tube pathology can lead to early detection of high-grade serous ovarian carcinomas and thus earlier management and therefore improved prognosis. In that setting, fallopian tube cytology can be a promising screening tool for high-risk patients, such as BRCA mutation carriers that have decided to postpone prophylactic hysterectomy and bilateral salpingoophorectomy and those women who have a positive family history. Current NCCN guidelines regarding these women include transvaginal ultrasound and serum CA-125 levels evaluation [11]. However, many experts believe that those methods alone are ineffective for discovering early ovarian cancer neither in high-risk women nor in the general population [12–15].

Our findings highlight the promising diagnostic ability of salpingeal cytology, since the cytologic evaluation of the smears in our study reached a high rate of detecting malignancy of the fallopian tube, including a case of serous tubal intraepithelial neoplasia (STIC). Additionally, based on the existing relative literature and on the principles of classical morphological fallopian tube cytology, we developed a rating system highlighting specific areas that have a significant effect in formulating the tubal cytological diagnosis. Our CytoSaLPs Score tool was also found to be of high diagnostic potential regarding the detection of fallopian tube malignancies, with an augmented specificity and positive predictive value (PPV) when compared with traditional cytology (Table 1). A meticulous scanning revealed no other report of a fallopian cytology evaluation method with such an exceptionally high accuracy. Based on our findings so far, the salpingeal smear of a patient with a CytoSaLPs Score lower than 16.5, is probably benign.

Until recently, the standard pathological evaluation of salpingeal tissue was based on a single section from the non-fimbriated part [16], while the SEE-Fim protocol is indicated in patients at risk for ovarian cancer [9]. However, the benefits and limitations, as well as the costs of applying the SEE-Fim protocol in all patients remains unclear. Additionally, the interpretation of the morphological features and alterations of the premalignant lesions is, until present, not standardized and requires a high level of expertise from the pathologists. On that basis, it can unavoidably be subjective. Currently, many authors have attempted to assess alternative methods of establishing the diagnosis of STIC. A recent study by Wu et al. analyzed fallopian tube samples from patients with benign diseases and HGSC patients with and without STIC, utilizing computational image analyses to distinguish alterations in stromal and epithelial cells. Interestingly, their findings suggest that STIC coexists with other morphological and topological changes which could be used as potential markers of STIC presence and indicate further pathological evaluation [17].

However, it has been suggested that the evaluation of salpingeal fimbriae should also be performed in low-risk patients in the setting of accidentally detecting occult fallopian tube lesions [18], although the majority of the studies have failed to detect malignant pathology in randomly selected fallopian tube specimens. Our study demonstrates that cytological sampling is efficient in detecting the presence of fallopian tube lesions and suggests that cytologic examination of the fimbriae in low-risk women too, could identify cases of early-stage tubal/ovarian/peritoneal malignancy that would otherwise have not been recognized.

Fallopian tube cytology has already been utilized in order to obtain cultures and establishing the diagnosis of salpingitis caused by Chlamydia [19, 20] and endometriosis, as it has been reported by Matsushima et al. [21]. None of those studies, however, has described normal or abnormal fallopian tube epithelium. Rodriguez et al. in their experimental study presented in 2013 was the first to meticulously describe the cytologic features from fallopian tube brush specimens and present a summary of the cytologic findings that can be observed [7]. Interestingly, in a following cytologic study.

of fallopian tube samples by Chen et al. in 2016, specific cytological features such as three-dimensional clusters and large cherry red nucleoli were most commonly corelated with a malignant cytological diagnosis [8].

Regarding the most efficient method of obtaining the fallopian tube smear, various approaches, both laparoscopic and hysteroscopic as well as in vivo and ex vivo have been described. Chen et al. showed that cytology-based diagnosis from ex-vivo obtained smears was 100% identical with the pathological diagnosis of HGSC [8]. Rodriguez et al. evaluated 15 fallopian tube samples, 10 of which were obtained laparoscopically, 4 hysteroscopically and 1 ex-vivo but illuminated the inefficacy of the hysteroscopic method in obtaining cytology samples from the infundibulum, resulting from the inability of the brush to insert further than 3-cm from the utero-tubal junction. The laparoscopic method showed better outcomes, although it is clear that undergoing a laparoscopic operation in order to evaluate the fallopian tubes is not acceptable as a screening method [22]. Impressively, Powell et al., introduced a novel hysteroscopic catheter the distal part of which was inserted through the utero-tubal junction and attached itself to the tubal epithelium with the aid of a built-in balloon [23]. The study included 50 women and the authors reported successful catheterization of the visible tubo-uterine junctions in 72% of the cases and adequate specimen samples were obtained in 68% of cases. Moreover, cytology diagnosis closely matched the final pathology report at a rate of 95%, deeming the aforementioned catheter as a very promising device.

Our method requires minimal effort and time beyond the standard pathological evaluation as well as minimized costs, although this could add up if applied to the general population. Based on the postulate of the tubal origin of the majority of pelvic cancers, there is now a growing trend to perform an opportunistic bilateral salpingectomy in women undergoing pelvic surgery for any indication, similar to the concept of incidental appendectomy [24]. This strategy can bear implications for discussion of implementation of population-level cytologic tube evaluation, during all benign gynecologic surgeries, and particularly those that do not result in salpingectomy, such as cesarean section, tubal sterilization, endometriosis surgery, diagnostic laparoscopy, appendectomy and any pelvic surgery in general, including minimal invasive procedures in an outpatient basis such as hysteroscopy.

However, there are some limitations and biases that need to be taken under consideration before drawing safe conclusions. First of all, our sample size, albeit the largest described in the literature so far, still remains too small to interpret or generalize our results. Moreover, our study is limited to a single-center experience and more specifically to a Gynecological Oncology Department, so that our patients’ pool includes women who already had increased risk for cancer (elevated CA-125 and HE4) and is restricted to be representative of women of all ages and health status. The PPV is indeed much higher in a cancer orientated population, nevertheless the population of our study includes cancer patients at a large percentage as well as women with benign condition. Moreover, our follow-up time is limited to assess if there is a correlation between our cytological findings and the development of extrauterine high grade serous carcinomas. Finally, from the perspective of early cancer detection, the size of this population is a determining parameter and the absence of randomized controlled trials is indeed a significant drawback in order to compare and establish our results. Furthermore, the sample retrieval method for the population-based screening test sets important technical limitations. We retrieved all our samples ex-vivo, since it is very difficult to retrieve in-vivo samples. The laparoscopic method is invasive and the hysteroscopic based approach did not give encouraging results so far as it is very difficult to take samples from the fimbriae. Clearly, ex-vivo sampling that are included in our study, cannot be applied as a screening tool as the organ of interest has already been excised. Thus, larger confirmatory studies are warranted so as to yield reliable and precise estimates, and in case that our results indicate satisfactory results regarding the diagnostic accuracy of salpingeal cytopathology, new technologies are demanded to create new scientific instruments that ensure a non-invasive obtainment-method that can be applied to the general population.

## Conclusion

Salpingeal cytology appears to be a highly sensitive, specific method to detect ovarian malignancies and possibly atypical lesions in the fallopian tubes. The present study indicates that the CytoSaLPs Score is reliable and of great value regarding the cytomorphologic evaluation of the fallopian tubes. Further prospective studies are needed to investigate the feasibility of in-vivo tubal smear collection, so as to establish.

The efficacy of our tool to deliver an early detection in women with a high-risk for ovarian/fallopian and primary peritoneal cancer, but also in women undergoing any gynecologic/pelvic surgery in the setting of incidental sampling.

## Electronic supplementary material

Below is the link to the electronic supplementary material.

Supplementary Material 1

## Data Availability

The datasets used and/or analysed during the current study available from the corresponding author on reasonable request.
